# Supplementary material for: Resolving the full spectrum of human genome variation using Linked-Reads
Source: Genome Res. 2019 Apr;29(4):635–45. doi: 10.1101/gr.234443.118 (PMC6442396; doi:10.1101/gr.234443.118)
Supplement: Supplemental Material [file supp_gr.234443.118_Supplemental_longranger-2.2.zip › longranger-2.2.tar.bz2!/lr2.2-public/lib/stan/cmdstan/stan/doc/api/html/math_2matrix_2typedefs_8hpp_source.html]

Stan: src/stan/math/matrix/typedefs.hpp Source File


|  |  |
| --- | --- |
|  | Stan  2.5.0  probability, sampling & optimization |


- Main Page
- Related Pages
- Namespaces
- Classes
- Files

- File List
- File Members

All Classes Namespaces Files Functions Variables Typedefs Enumerations Enumerator Friends Macros Pages

- src
- stan
- math
- matrix

typedefs.hpp

Go to the documentation of this file.

1 #ifndef STAN\_\_MATH\_\_MATRIX\_\_TYPEDEFS\_HPP

2 #define STAN\_\_MATH\_\_MATRIX\_\_TYPEDEFS\_HPP

3

4 #include <stan/math/matrix/Eigen.hpp>

5 #include <stan/math/matrix/meta/index\_type.hpp>

6

7 namespace stan {

8

9  namespace math {

10

14  typedef

15  index\_type<Eigen::Matrix<double,Eigen::Dynamic,Eigen::Dynamic> >::type

16  size\_type;

17

21  typedef

22  Eigen::Matrix<double,Eigen::Dynamic,Eigen::Dynamic>

23  matrix\_d;

24

28  typedef

29  Eigen::Matrix<double,Eigen::Dynamic,1>

30  vector\_d;

31

35  typedef

36  Eigen::Matrix<double,1,Eigen::Dynamic>

37  row\_vector\_d;

38

39  }

40 }

41

42 #endif

stan::math::matrix\_d

Eigen::Matrix< double, Eigen::Dynamic, Eigen::Dynamic > matrix\_d

Type for matrix of double values.

**Definition:** typedefs.hpp:23

index\_type.hpp

Eigen.hpp

stan::math::size\_type

index\_type< Eigen::Matrix< double, Eigen::Dynamic, Eigen::Dynamic > >::type size\_type

Type for sizes and indexes in an Eigen matrix with double e.

**Definition:** typedefs.hpp:16

stan::math::vector\_d

Eigen::Matrix< double, Eigen::Dynamic, 1 > vector\_d

Type for (column) vector of double values.

**Definition:** typedefs.hpp:30

stan::math::row\_vector\_d

Eigen::Matrix< double, 1, Eigen::Dynamic > row\_vector\_d

Type for (row) vector of double values.

**Definition:** typedefs.hpp:37


---

[ Stan Home Page ]

*© 2011–2014,
Stan Development Team.*
